# Racial/Ethnic Disparities in Fatal Unintentional Drowning Among Persons Aged ≤29 Years — United States, 1999–2010

**Published:** 2014-05-16

**Authors:** Julie Gilchrist, Erin M. Parker

**Affiliations:** 1 Division of Unintentional Injury Prevention, National Center for Injury Prevention and Control, CDC

In the United States, almost 4,000 persons die from drowning each year (1). Drowning is responsible for more deaths among children aged 1–4 years than any other cause except congenital anomalies (2). For persons aged ≤29 years, drowning is one of the top three causes of unintentional injury death (2). Previous research has identified racial/ethnic disparities in drowning rates (3,4). To describe these differences by age of decedent and drowning setting, CDC analyzed 12 years of combined mortality data from 1999–2010 for those aged ≤29 years. Among non-Hispanics, the overall drowning rate for American Indians/Alaska Natives (AI/AN) was twice the rate for whites, and the rate for blacks was 1.4 times the rate for whites. Disparities were greatest in swimming pools, with swimming pool drowning rates among blacks aged 5–19 years 5.5 times higher than those among whites in the same age group. This disparity was greatest at ages 11–12 years; at these ages, blacks drown in swimming pools at 10 times the rate of whites. Drowning prevention strategies include using barriers (e.g., fencing) and life jackets, actively supervising or lifeguarding, teaching basic swimming skills and performing bystander cardiopulmonary resuscitation (CPR). The practicality and effectiveness of these strategies varies by setting; however, basic swimming skills can be beneficial across all settings.

Death certificate data for persons aged ≤29 years for 1999–2010 were obtained from the National Vital Statistics System* to identify persons who had died from unintentional drowning. Fatal unintentional drowning was defined as any death for which the underlying cause included any of the following codes from the *International Classification of Diseases, 10th Revision*: W65–W74, V90, or V92. By international standards, boating-related drowning (V90 and V92) is classified as a transportation-related death. However, most boating in the United States is not for the purpose of transportation; therefore, drowning while boating is included in this report. Drowning was examined by setting (bathtub, swimming pool, natural water, boating, and other or unspecified), age, and race/ethnicity. Race/ethnicity was coded into five mutually exclusive categories: Hispanic (of any race), and four non-Hispanic racial groups (white, black, AI/AN, and Asian/Pacific Islander (A/PI)). Age was divided into 5-year age groups for overall and setting-specific drowning deaths among each racial/ethnic category. Among blacks, whites, and Hispanics, overall drowning was presented by year of age, and drowning in swimming pools and natural water were categorized by 2-year age groups to provide stable rates after infancy. Rates of drowning death for infants aged <1 year were dissimilar from other ages and were not combined. Death rates per 100,000 population were calculated using 1999–2010 U.S. Census bridged-race population estimates. Differences between rates representing at least 100 deaths were determined using z-tests; rates based on fewer than 100 deaths were compared using 95% confidence intervals from a gamma distribution.

Among all settings combined, AI/AN aged ≤29 years had the highest rates of drowning, with blacks having the second highest rates (Table). Overall, the rate for AI/AN was twice the rate for whites (2.57 per 100,000 population versus 1.32, respectively) and the rate for blacks was 1.4 times the rate for whites (1.90 versus 1.32, respectively). When considering drowning rates by age group, AI/AN were not statistically different from other races for some age groups (whites at ages 1–4 years, blacks at ages 5–9, 10–14, and 15–19 years). Among all settings combined, rates among A/PI aged ≤29 years were lower than for other groups; however, A/PI rates were higher than for whites and Hispanics at ages 5–9 years and higher than for whites at ages 10–14 and 15–19 years. By setting, disparities in drowning rates were greatest for swimming pool deaths, where the drowning death rate for blacks aged 5–19 years was 5.5 times the rate for whites (0.55 per 100,000 population versus 0.10, respectively).

Among each racial/ethnic group, drowning settings varied similarly by age group (Table). Infants aged <1 year most commonly drowned in bathtubs, accounting for 62.5% (435 of 696) of drowning at this age. Children aged 1–4 and 5–9 years most commonly drowned in swimming pools, accounting for 51.4% (2,852 of 5,547) and 33.9% (616 of 1,818), respectively. The older age groups most commonly drowned in natural water settings.

Racial/ethnic differences in overall drowning rates varied by each year of age (Figure 1). The highest rates for all three groups presented were among children aged 1 year, with rates for whites (5.22 per 100,000 population) higher than those for Hispanics (4.14), and rates for Hispanics higher than those for black children (2.98). Between the ages of 1 year and 5 years, drowning rates decreased significantly for each racial/ethnic group (83% for whites, 85% for Hispanics, and 43% for blacks). However, the drowning rates for black children were significantly higher than those for whites and Hispanics at every age from 5 years through 18 years. The greatest disparity for blacks compared with whites and Hispanics was at age 10 years (rate ratios of 4.2 and 5.3, respectively).

For drowning in swimming pool settings, the rates for black, white, and Hispanic children aged 1–2 years were highest; pool drowning rates among whites (2.53 per 100,000 population) were significantly higher than those for Hispanics (1.85) and blacks (1.59) in this age group. Rates of pool drowning among blacks were significantly higher than those for whites for ages 5–6 through 27–28 years and higher than those for Hispanics for ages 3–4 through 19–20 years; rate ratios were highest at ages 11–12 years for both comparisons (10.4 and 6.4, respectively) (Figure 2).

For drowning in natural water settings, the rates for blacks were significantly higher than those for whites for ages 7–8 through 17–18 years and higher than those for Hispanics for ages 5–6 through 15–16 years; rate ratios were highest at 13–14 years for both comparisons (3.5 and 2.6, respectively) (Figure 2). Rates of drowning in natural water settings among Hispanics were similar to those among whites from 5–6 years through 15–16 years, when rates among Hispanics increased, peaking at 1.35 per 100,000 population among Hispanics aged 19–20 years.

## Discussion

Identifying racial/ethnic drowning disparities by setting can help focus prevention efforts. For instance, swimming pools are generally considered safer than natural water venues for aquatic activities because their depth is known and bottom often visible, they lack currents and underwater hazards, and the side can be reached a relatively short distance away. However, in the United States, drowning in a swimming pool continues to be a major threat to the health of toddlers and preschool children (1,4). Moreover, swimming pool drowning rates for black children, adolescents, and young adults were elevated compared with those for other racial/ethnic groups. Research suggests that learning basic swimming skills (e.g., controlled breathing, floating, and traversing a distance) can reduce drowning risks (5,6); however, many children and adults, especially blacks, report limited swimming skills (7,8).

What is already known on this topic?

Drowning is the leading cause of unintentional injury death among children aged 1–4 years and one of the top three causes among persons aged ≤29 years. Rates of drowning among some racial/ethnic groups (e.g., non-Hispanic blacks and American Indians/Alaska Natives) are higher than rates for non-Hispanic whites. Black children and adults also report having more limited swimming ability than whites.

What is added by this report?

This is the first report to examine racial/ethnic disparities in fatal drowning rates by age and setting. Overall, American Indians/Alaska Natives were twice as likely, and blacks 1.4 times as likely, to drown as whites. The disparity increased when only drowning deaths in swimming pools were considered. Blacks aged 5–19 years were 5.5 times more likely to drown in a swimming pool than their white peers, and at ages 11–12 years, blacks drowned in swimming pools at 10 times the rate of whites.

What are the implications for public health practice?

Swimming skills can be life-saving. The disparity in self-reported swimming skills among black children and adults might help to explain the disparity in drowning rates and should be addressed through support of swimming lessons and other proven interventions.

Among all racial/ethnic groups, rates of drowning in natural water settings increase among teens and young adults. Alcohol use and increased independence, with resulting reduced supervision, might play a role in these deaths (9). In these locations, otherwise effective interventions such as fences and lifeguarding might not be feasible, but basic swimming skills might reduce drowning risk when teens or young adults enter the water, whether intentionally or unintentionally (6).

The high drowning rates among AI/AN populations reported here, especially in natural water settings, are consistent with previous studies (10). AI/AN children, teenagers, and young adults might be at higher risk because of greater exposure to natural bodies of water (10); little is known about AI/AN swimming skills.

Lack of exposure data is a major limitation in epidemiologic studies of drowning. For instance, in this study, drowning in a swimming pool was almost six times more likely among black children and adolescents aged 5–18 years than among their white peers. However, if a group’s exposure to pools is less than that of their peers, their true drowning risks, based on equivalent exposure, could be even higher. The extent of exposure to recreational water settings likely varies substantially by age, sex, season, level of swimming skill, and other factors. Because exposure data are not available, the rates reported are population-based.

Additionally, the lack of critical information on death certificates limits more detailed analyses to explore causes of disparities. Death certificates do not include details on known risk and protective factors such as the victim’s activities and swimming skill, the body of water, weather conditions, health conditions, use of life jackets, type and functionality of fences or barriers, supervision type and quality (e.g., impaired), presence of lifeguards, alcohol use, and whether CPR was performed by a bystander. These measures could be used to further explain disparities and would be helpful to guide targeted prevention programs.

Drowning continues to be a public health problem affecting racial/ethnic groups disparately among different age groups and in different aquatic settings; these differences require implementation of multiple prevention strategies. Drowning prevention strategies include use of barriers (like fencing) and life jackets, actively supervising or lifeguarding, teaching basic swimming skills, and performing bystander CPR. Practicality and effectiveness of these strategies might vary in different settings; however, basic swimming skills can be beneficial across all settings. Racial/ethnic minorities should be encouraged and enabled to gain skills needed to survive in the water. Additional information regarding drowning risk factors and prevention strategies is available at http://www.cdc.gov/homeandrecreationalsafety/water-safety/index.html.

## Figures and Tables

**FIGURE 1:**
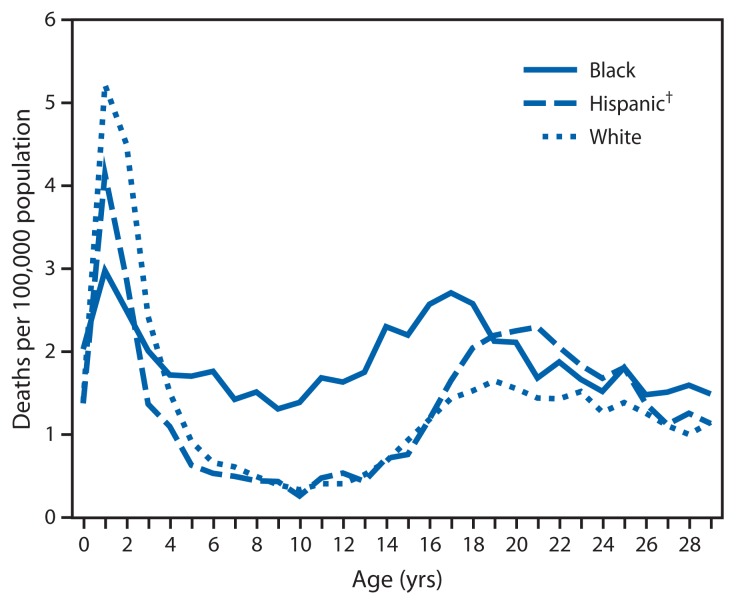
Rates of fatal unintentional drowning among persons aged ≤29 years, by age and race/ethnicity* — United States, 1999–2010 * Rates for other racial/ethnic groups are excluded because rates are not stable for single year of age. ^†^ Persons identified as Hispanic might be of any race. Persons identified as white or black are all non-Hispanic.

**FIGURE 2:**
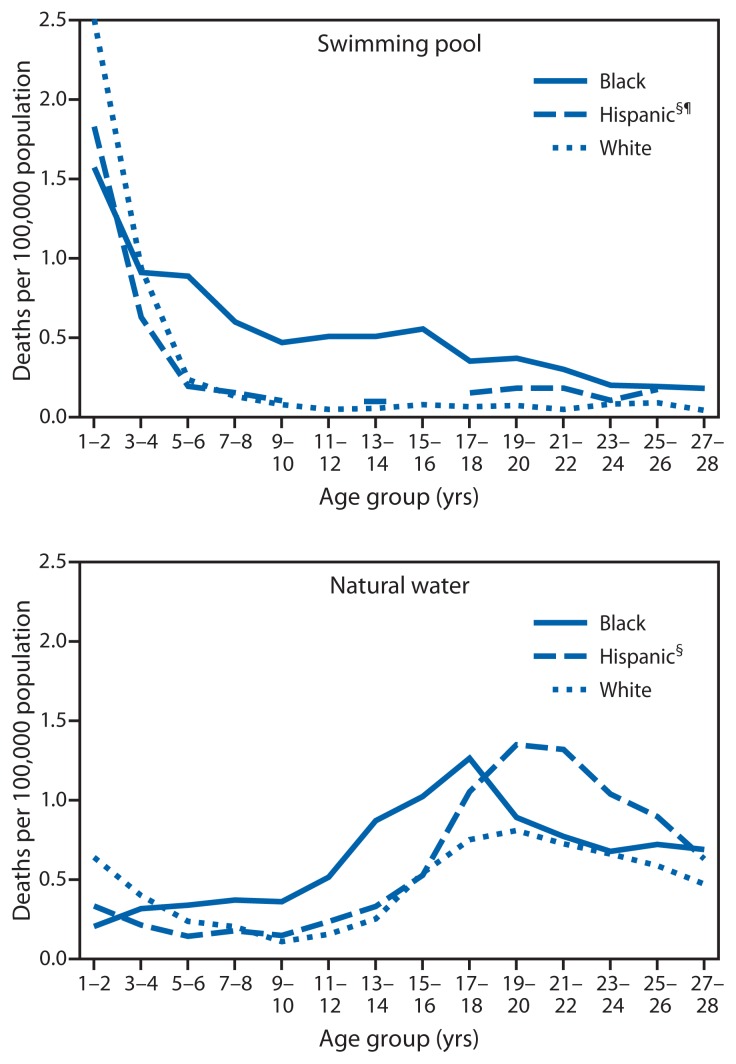
Rates of fatal unintentional drowning in swimming pools and natural water settings among persons aged 1–28 years,* by age group and race/ethnicity^†^ — United States, 1999–2010 * Rates for infants aged <1 year are dissimilar to all others and are excluded. Remaining ages 1–28 years are paired to create stable rates. ^†^ Rates for other racial/ethnic groups are excluded because rates were not stable. ^§^ Persons identified as Hispanic might be of any race. Persons identified as white or black are all non-Hispanic. ^¶^ Death rates based on <20 deaths suppressed for unreliability.

**TABLE:** Numbers and rates* of fatal unintentional drowning among persons aged ≤29 years, by setting, race/ethnicity, and age group — United States, 1999–2010

	Age group (yrs)															
	
	<1		1–4		5–9		10–14		15–19		20–24		25–29		Total	
								
Setting	No.	Rate	No.	Rate	No.	Rate	No.	Rate	No.	Rate	No.	Rate	No.	Rate	No.	Rate
**Drowning (all settings)**																
AI/AN	11	—†	73	3.83	37	1.50	33	1.22	73	2.63	77	3.25	75	3.68	**379**	**2.57**
Asian/Pacific Islander	—	—	127	1.42	91	0.84	72	0.67	206	1.80	179	1.38	162	1.10	**846**	**1.18**
Black	145	2.03	654	2.30	569	1.54	690	1.75	956	2.44	620	1.78	496	1.58	**4,130**	**1.90**
Hispanic§	156	1.38	1,013	2.40	246	0.51	224	0.48	709	1.57	932	2.02	619	1.34	**3,899**	**1.37**
White	371	1.39	3,665	3.40	865	0.61	712	0.47	2,108	1.35	2,166	1.45	1,694	1.18	**11,581**	**1.32**
**Total**¶	**696**	**1.46**	**5,547**	**2.93**	**1,818**	**0.76**	**1,737**	**0.69**	**4,064**	**1.59**	**3,988**	**1.62**	**3,062**	**1.29**	**20,912**	**1.43**
**Swimming pool**																
AI/AN	—	—	13	—	—	—	—	—	—	—	—	—	—	—	**31**	**0.21**
Asian/Pacific Islander	—	—	69	0.77	35	0.32	16	—	23	0.20	26	0.20	44	0.30	**213**	**0.30**
Black	—	—	357	1.26	251	0.68	205	0.52	177	0.45	95	0.27	61	0.19	**1,152**	**0.53**
Hispanic§	—	—	531	1.26	82	0.17	42	0.09	61	0.14	71	0.15	61	0.13	**854**	**0.30**
White	48	0.18	1,872	1.74	233	0.17	87	0.06	121	0.08	97	0.06	100	0.07	**2,558**	**0.29**
**Total**¶	**61**	**0.13**	**2,852**	**1.51**	**616**	**0.26**	**356**	**0.14**	**383**	**0.15**	**295**	**0.12**	**268**	**0.11**	**4,831**	**0.33**
**Natural water**																
AI/AN	—	—	26	1.36	16	—	21	0.77	40	1.44	41	1.73	36	1.77	**180**	**1.22**
Asian/Pacific Islander	—	—	24	0.27	28	0.26	38	0.35	120	1.05	105	0.81	82	0.56	**397**	**0.55**
Black	—	—	74	0.26	134	0.36	244	0.62	427	1.09	267	0.77	217	0.69	**1,363**	**0.63**
Hispanic§	—	—	116	0.28	79	0.16	115	0.25	413	0.92	553	1.20	339	0.73	**1,616**	**0.57**
White	—	—	559	0.52	279	0.20	280	0.18	1,080	0.69	1,058	0.71	753	0.53	**4,015**	**0.46**
**Total**¶	**—**	**—**	**800**	**0.42**	**539**	**0.23**	**700**	**0.28**	**2,083**	**0.82**	**2,031**	**0.82**	**1,433**	**0.60**	**7,594**	**0.52**
**Boating**																
AI/AN	—	—	—	—	—	—	—	—	—	—	12	—	—	—	**34**	**0.23**
Asian/Pacific Islander	—	—	—	—	—	—	—	—	12	—	—	—	—	—	**30**	**0.04**
Black	—	—	—	—	12	—	14	—	41	0.10	48	0.14	42	0.13	**160**	**0.07**
Hispanic§	—	—	—	—	—	—	10	—	25	0.06	51	0.11	50	0.11	**144**	**0.05**
White	—	—	22	0.02	45	0.03	74	0.05	216	0.14	303	0.20	256	0.18	**920**	**0.10**
**Total**¶	**—**	**—**	**28**	**0.01**	**65**	**0.03**	**101**	**0.04**	**302**	**0.12**	**423**	**0.17**	**367**	**0.15**	**1,291**	**0.09**
**Bathtub**																
AI/AN	—	—	—	—	—	—	—	—	—	—	—	—	—	—	**19**	**—**
Asian/Pacific Islander	—	—	—	—	—	—	—	—	—	—	—	—	—	—	**24**	**0.03**
Black	88	1.23	79	0.28	16	—	23	0.06	16	—	25	0.07	31	0.10	**278**	**0.13**
Hispanic§	107	0.94	107	0.25	12	—	12	—	13	—	12	—	18	—	**281**	**0.10**
White	226	0.85	299	0.28	56	0.04	63	0.04	81	0.05	150	0.10	135	0.09	**1,010**	**0.12**
**Total**¶	**435**	**0.91**	**500**	**0.26**	**87**	**0.04**	**99**	**0.04**	**113**	**0.04**	**190**	**0.08**	**191**	**0.08**	**1,615**	**0.11**
**Other or unspecified**																
AI/AN	—	—	28	1.47	10	—	—	—	24	0.86	20	0.84	23	1.13	**115**	**0.78**
Asian/Pacific Islander	—	—	26	0.29	24	0.22	16	—	49	0.43	36	0.28	27	0.18	**182**	**0.25**
Black	50	0.70	142	0.50	156	0.42	204	0.52	295	0.75	185	0.53	145	0.46	**1,177**	**0.54**
Hispanic§	42	0.37	256	0.61	68	0.14	45	0.10	197	0.44	245	0.53	151	0.33	**1,004**	**0.35**
White	87	0.33	913	0.85	252	0.18	208	0.14	610	0.39	558	0.37	450	0.31	**3,078**	**0.35**
**Total**¶	**187**	**0.39**	**1,367**	**0.72**	**511**	**0.21**	**481**	**0.19**	**1,183**	**0.46**	**1,049**	**0.43**	**803**	**0.34**	**5,581**	**0.38**

**Abbreviation:** AI/AN = American Indian/Alaska Native.

* Per 100,000 population.

† Death counts based on <10 deaths suppressed for confidentiality. Death rates based on <20 deaths suppressed for unreliability.

§ Persons identified as Hispanic might be of any race. Persons identified in the categories of white, black, AI/AN, or Asian/Pacific Islander are all non-Hispanic.

¶ Total rates for each setting include “not stated” race/ethnicity.

## Footnotes

* Additional information available at http://wonder.cdc.gov/ucd-icd10.html.
